# Shared mediators, divergent networks: a tissue-response hypothesis for discordant phenotypes in chronic spontaneous urticaria and atopic dermatitis

**DOI:** 10.3389/falgy.2026.1976519

**Published:** 2026-10-12

**Authors:** Young-Min Ye, Michihiro Hide

**Affiliations:** 1 Department of Allergy and Clinical Immunology, Ajou University School of Medicine, Suwon, Republic of Korea; 2 Department of Dermatology, Hiroshima City Hiroshima Citizens Hospital, Hiroshima City Hospital Organization, Hiroshima, Japan; 3 Department of Dermatology, Graduate School of Biomedical and Health Sciences, Hiroshima University, Hiroshima, Japan

**Keywords:** atopic dermatitis, chronic spontaneous urticaria, complement-coagulation crosstalk, mast cells, MRGPRX2, neuro-immune inflammation, platelet-activating factor, vascular permeability

## Abstract

Chronic spontaneous urticaria (CSU) and atopic dermatitis (AD) are pruritic inflammatory skin diseases that share partly overlapping mast-cell-related pathways, including IgE/FcεRI signaling, type 2 inflammation, neuroimmune signaling, and lipid mediator pathways. Yet they usually produce distinct phenotypes: transient wheals and angioedema in CSU and persistent eczematous lesions with inflammation in AD. Whether disease activity in the two conditions fluctuates concordantly or independently within the same patient has not been directly tested. This Perspective proposes that phenotypic divergence reflects the tissue-response network in which mast cell activation is embedded rather than mediator identity alone. In CSU, autoimmune and autoallergic FcεRI-dependent activation, MRGPRX2-related neuropeptide pathways, coagulation/fibrinolysis-C5a signaling, and platelet-activating factor (PAF)/lipid mediators may preferentially converge on mast cells and basophils poised for rapid degranulation within the dermal vascular/perivascular unit. This network favors histamine bursts, endothelial hyperpermeability, neurovascular itch, and feedback-limited wheal formation. In AD, mast cells are embedded in a barrier-disrupted, alarmin-rich neuroepidermal niche, producing a multicellular inflammatory network involving keratinocyte activation, cytokine and chemokine production, neuronal sensitization, itch-scratch cycling, and tissue remodeling, although degranulation and vascular effects may also occur. Neuropeptides and PAF illustrate how shared mediators may be preferentially routed toward vascular leakage in CSU or neuroepidermal inflammatory amplification in AD. This tissue-addressed mast cell output model is proposed as a testable hypothesis for how overlapping inflammatory mediators may generate feedback-limited vascular whealing in CSU but sustained neuroepidermal inflammation in AD. The model emphasizes that shared pathways are preferentially weighted toward different tissue-level outcomes in CSU and AD.

## Introduction

Chronic spontaneous urticaria (CSU) and atopic dermatitis (AD) are common chronic inflammatory skin diseases characterized by severe pruritus and recurrent or persistent skin symptoms (1, 2). Both diseases impair sleep, daily functioning, quality of life, and psychological well-being, and are associated with psychiatric comorbidities, systemic comorbidities, and substantial socioeconomic burden (2, 3). Clinically, eczema-dominant AD and wheal-dominant CSU are often recognized as distinct patterns, even when overlapping inflammatory pathways or comorbid disease are present. Both diseases share itch, IgE/FcεRI signaling, mast cells, type 2 inflammation, non-IgE mast-cell receptors, lipid mediators, and neural input.

Epidemiologic data support a robust, and at least partly bidirectional, association between AD and CSU (4–9). Reported AD prevalence among CSU patients varies with study design, diagnostic criteria, and population, ranging broadly from approximately 10%–40% and consistently exceeding rates in matched controls or the general population (4–6). Several cohorts additionally report the risk of AD to be approximately 2- to 5-fold higher among CSU patients than matched controls (7–10). A history of AD has also been linked to increased subsequent risk of CSU, particularly when AD onset precedes CSU by several years (7, 9). Together, these findings demonstrate comorbidity and, in some cohorts, a temporal sequence of onset; they do not establish temporal dissociation of disease activity, because longitudinal studies concurrently measuring validated CSU and AD activity indices in the same patients are lacking. Our clinical impression is that severe eczema and severe wheals do not commonly peak together in the same patient. We treat this observation as a hypothesis to be tested and propose that, when present, it may reflect how similar upstream mediators are interpreted differently according to local tissue context and systemic background.

As a Perspective focused on a mast-cell-centered comparison rather than a comprehensive review of either disease's pathogenesis, this article develops a specific hypothesis: how overlapping mast-cell-related signals may be preferentially translated into transient vascular whealing in CSU or continuous eczematous inflammation in AD (Figure 1). The greater mechanistic detail devoted to CSU below reflects the specific scope of this model and should not be read as implying that AD is primarily a mast-cell-driven disease or that the pathways discussed comprehensively represent AD pathogenesis. We propose that CSU and AD are best understood as distinct tissue-response programs. In CSU, mast-cell activation is wired to the dermal vascular/perivascular unit, producing edema, wheal, flare, and angioedema. In AD, mast-cell activation is embedded in a barrier-disrupted epithelial-alarmin-neuro-immune niche, producing chronic itch, scratching, keratinocyte activation, and eczematous inflammation. This Perspective develops a comparative tissue-response model that distinguishes feedback-limited vascular whealing from continuous neuro-epidermal inflammation (Table 1).

**Figure 1:**
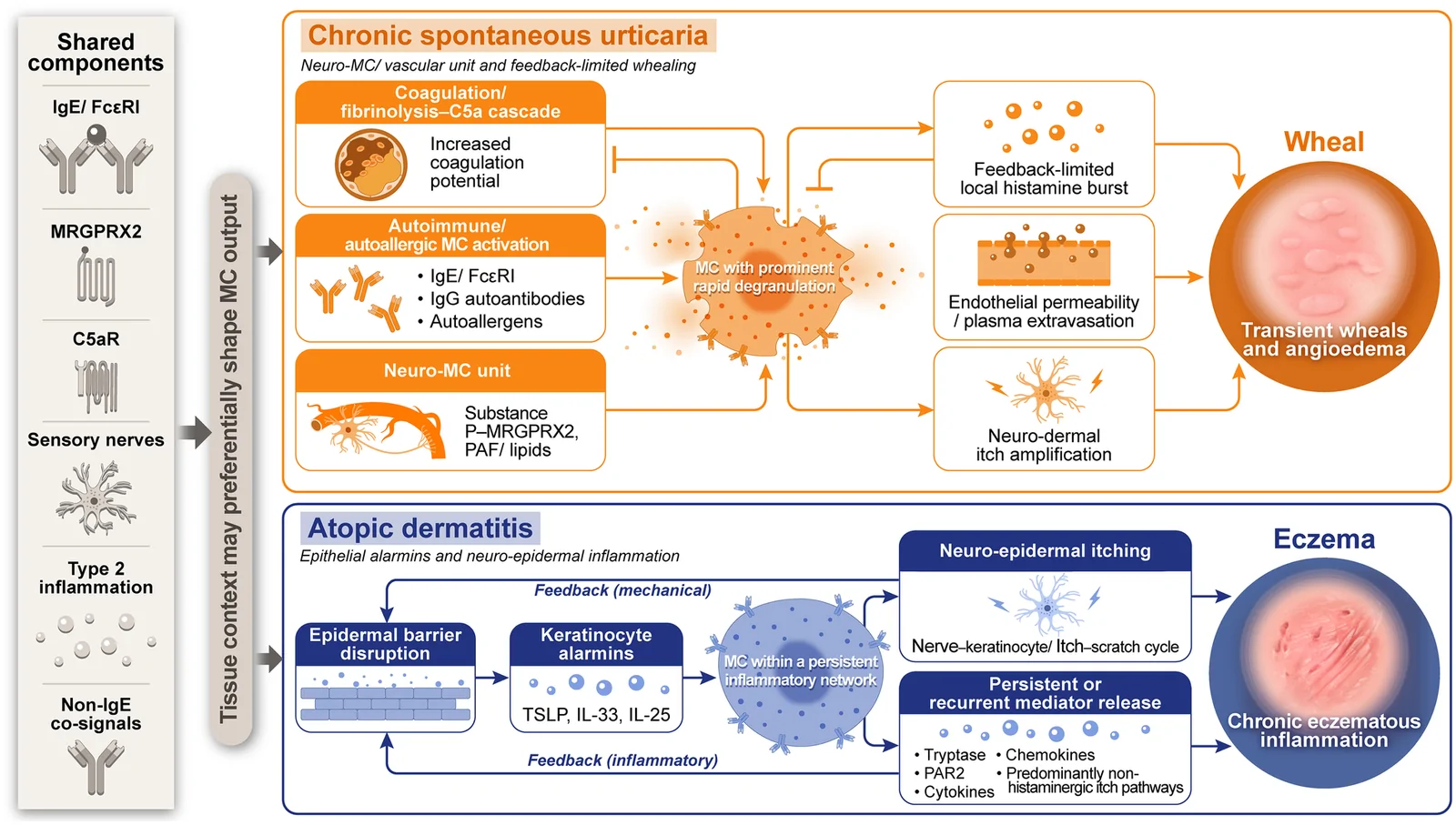
Proposed mast-cell-centered tissue-addressed model of chronic spontaneous urticaria (CSU) and atopic dermatitis (AD). This schematic presents a mast-cell-centered conceptual model rather than a comprehensive comparison of CSU and AD pathogenesis. CSU and AD engage partly overlapping mast-cell-related pathways, including IgE/FcεRI signaling, MRGPRX2, C5aR, itch/sensory nerves, type 2 inflammation, and non-IgE co-signals. In CSU, autoimmune/autoallergic mast-cell activation, coagulation/fibrinolysis-C5a signaling, neuro-mast cell-vascular interactions, and the PAF/lipid-mediator pathways may converge within the dermal vascular/perivascular unit. This network is proposed to favor rapid degranulation-associated output, local and short-term histamine bursts, plasma extravasation, neurovascular itch, and transient wheal formation, although cytokine release may also accompany mast-cell activation. PAF may additionally contribute to vascular leakage without necessarily requiring detectable mast-cell degranulation, with possible focal mast-cell amplification under selected conditions. In AD, epidermal barrier disruption and keratinocyte-derived alarmins condition mast cells alongside multiple resident and infiltrating cell populations within a neuroepidermal inflammatory niche. In this context, mast-cell outputs, including degranulation-associated mediators, cytokines, proteases, and chemokines, may be incorporated into keratinocyte activation, neuronal sensitization, non-histaminergic itch, and sustained eczematous inflammation. Mechanical feedback through scratching and inflammatory feedback through recurrent mediator release may reinforce barrier disruption and alarmin signaling. The illustrated patterns represent a proposed relative weighting of overlapping pathways rather than mechanisms exclusive to either disease. AD, atopic dermatitis; C5aR, complement component 5a receptor; CSU, chronic spontaneous urticaria; FcεRI, high-affinity IgE receptor; MRGPRX2, mas-related G protein-coupled receptor X2; PAF, platelet-activating factor.

**Table 1:** Mast cell-centered comparison of tissue-response networks in CSU and AD.

Dimension	Chronic spontaneous urticaria	Atopic dermatitis
Dominant tissue-response network	Dermal neuro-MC/vascular unit coupled to endothelial permeability, plasma extravasation, wheal formation, and angioedema	Barrier-disrupted epithelial-alarmin andneuronal niche coupled to itch, inflammation, and remodeling
Proposed mast-cell functional weighting	Weighted toward rapid deranulation-associated vascular output, favoring local histamine bursts and vascular leakage; cytokine release may also occur	Conditioned toward inflammatory and neuro-immune amplification while retaining the capacity for degranulation, protease release, and vascular effects
Major upstream inputs	FcεRI aggregation, IgG autoantibodies, IgE autoallergens, MRGPRX2, coagulation/fibrinolysis-C5a, neuropeptides, PAF/lipid mediators	Barrier disruption, TSLP, IL-33, IL-25, IL-4/IL-13, IL-31, scratching, microbial/allergen proteases, PAR2, IgE/FcεRI priming, neuropeptides, and lipid mediators
Neuropeptide-associated network	Neuro-dermal MC/vascular axis: SP-MRGPRX2 and other neuropeptide pathways may amplify MC activation, histamine release, vascular leakage, and wheal formation	Neuro-epidermal itch-scratch circuit: neuropeptides interact with keratinocytes, alarmins, type 2 cytokines, mast cells, and sensory nerves
PAF/lipid mediator-associated network	PAF may contribute to vascular/perivascular leakage without necessarily requiring detectable MC degranulation; focal MC amplification may occur under selected conditions	Elevated circulating PAF has been reported, but its cellular routing and tissue-level effects remain poorly defined
Temporal pattern of mediator output	Predominantly acute, burst-like, spatially restricted, and feedback-limited, although inflammatory mediator release may accompany degranulation	Recurrent or sustained inflammatory and neuro-immune signaling; often non-histaminergic itch pathway-dominant
Predominant tissue consequence	Transient vascular edema, generally without persistent tissue remodeling	Chronic eczematous inflammation, scratching injury, barrier dysfunction, and remodeling
Predominant clinical phenotype	Transient wheals, flare, angioedema, and rapid-onset itch	Chronic itch, Persistent or recurrent eczema, excoriation, and lichenification

AD, atopic dermatitis; C5a, complement component 5a; CSU, chronic spontaneous urticaria; FcεRI, high-affinity IgE receptor; MC, mast cell; MRGPRX2, mas-related G protein-coupled receptor X2; PAF, platelet-activating factor; PAR2, protease-activated receptor 2; SP, substance P; TSLP, thymic stromal lymphopoietin.

## Shared inflammatory modules do not necessarily produce shared skin lesions

CSU and AD share a common cellular substrate, the mast cell (2, 11). Both diseases draw on an overlapping repertoire of activating and degranulating inputs, including IgE/FcεRI signaling, Mas-related G protein-coupled receptor X2 (MRGPRX2)-related neuropeptide engagement, complement activation, itch-generating sensory input, type 2 cytokines, and non-IgE co-signals such as PAF and other lipid mediators (2, 12–17). These inputs are not uniformly weighted, either between the two diseases or within them. Their relative contribution varies by endotype. In CSU, this is reflected in the distinction between autoallergic (type I) and autoimmune (type IIb) subtypes (18, 19). In AD, cytokine-axis-based endotyping distinguishes type 2-dominant from non-type 2-skewed patterns, including Th1-, Th17-, and Th22-associated inflammation, which also vary by age and ancestry (16).

Yet shared mediators do not obviously explain why CSU produces wheals whereas AD produces eczema. If mediator identity alone determined phenotype, high mediator burden might be expected to produce phenotypic convergence. Our clinical impression that severe wheal and severe eczema do not commonly peak concurrently, even in patients with documented comorbid disease, has not been formally tested using concurrent validated activity measures. We therefore present temporal dissociation as a testable observation rather than an established phenomenon and hypothesize that the tissue-response network surrounding mast cells contributes to phenotypic expression.

## CSU: mast-cell output addressed to the dermal vascular/perivascular unit

CSU may be viewed as a dermal mast cell-vascular disease. Degranulation-ready mast cells and basophils are positioned within or near the microvascular unit (11, 20). Here, FcεRI aggregation, IgG autoantibodies against IgE or FcεRI, IgE autoantibodies to self-antigens, MRGPRX2 ligands, coagulation/fibrinolysis-C5a signals, neuropeptides, and lipid mediators can converge (12). The preferential tissue-level output is transient vascular escape characterized by local histamine release, endothelial hyperpermeability with resultant plasma extravasation, wheal formation, angioedema, and rapid resolution.

Of note, the threshold of histamine to induce visible flare (0.3–1 µM) is much less than that to induce wheal formation (3–10 µM) (21), suggesting that wheal formation depends on a distinct, higher-threshold mechanism specific to urticaria, rather than the lower-threshold neurogenic reflex underlying flare. Mathematical modeling of CSU supports this view (22). This work divides the wheal lifecycle into onset, development, and disappearance phases. Onset is driven by basophil-related endovascular positive feedback including the production of C5a from the plasma complement C5 (23). This step is triggered by small stimuli on basophils and/or mast cells, such as IgG autoantibodies against IgE/FcεRI, and/or autoantigens binding to IgE, or tissue factor-expressing monocytes, and depends on the increase of exogenous coagulation potential. Development is related to mast cell-mediated vascular gap formation. Wheal disappearance is governed by an autoinhibitory purinergic mechanism. Adenosine, generated through ATP breakdown in association with histamine release, suppresses further histamine release and tissue factor expression (23). This terminates the positive-feedback loop that drives wheal expansion. Consistent with this self-limiting dynamic, histamine release appears in a short burst. It is further diluted by the plasma extravasation it helps generate. Together, these dynamics explain the rapid resolution characteristic of wheals.

Neuropeptides and lipid mediators may extend this vascular circuit beyond mast-cell histamine alone (24). Substance P (SP) can activate skin mast cells through MRGPRX2 and has also been linked to NK1R-mediated vasodilation and plasma extravasation, thereby coupling sensory nerve input to dermal mast cell-vascular unit (25). Of note, both C5a and SP induce mast cell degranulation in the skin, but mast cells of other tissues show little or no responsiveness to these stimuli (26, 27). Moreover, SP induces rapid relase of TNF-α and IL-8 from human skin mast cells, but no significant levels of IL-4, IL-5, or IL-13 (28, 29). The release of mediators from human basophils induced by C5a largely depends on the environment, especially IL-3. C5a alone induces rapid release of histamine, but little or no production of IL-4 by human basophils, despite promoting vigorous histamine release (30). However, basophils pretreated with IL-3 show large and long-lasting release of IL-4, IL-13 and LTC4 in response to C5a (31). Thus, in the absence of IL-3, the activation of basophils and mast cells by C5a, SP or transient IgE/FcεRI engagement mainly releases histamine with little type 2 cytokine production. In the presence of IL-3, however, the same stimuli, particularly C5a on basophils and IgE/FcεRI on skin mast cells, can additionally drive type 2 cytokine release, favoring progression toward type 2 skin inflammation.

The role of PAF in this circuit is less settled. Human skin microdialysis studies found that PAF induces wheal-and-flare reactions without a measurable increase in dermal histamine or PGD2 release, unlike codeine-induced mast-cell activation (32). This has led to the proposal that PAF drives endothelial hyperpermeability independently of mast-cell degranulation. However, *in vitro* studies show that PAF can directly induce degranulation of human mast cells, including LAD2 cells and induced pluripotent stem cell-derived mast cells (33). We suggest these findings need not conflict. However, if PAF triggers both mast-cell degranulation together with rapid endothelial leakage, plasma extravasation could dilute or disperse locally released histamine below the detection threshold of microdialysis sampling. This is consistent with the burst-and-dilution dynamics. This proposed reconciliation is a hypothesis that has not been directly tested and would require simultaneous measurement of histamine, degranulation markers, and vascular permeability following PAF challenge.

Together, these mechanisms explain why CSU lesions arise abruptly and resolve without persistent epidermal remodeling. The wheal, in this framework, is a feedback-regulated vascular event rather than a continuously expanding inflammatory plaque.

## AD: mast-cell output addressed to the neuroepidermal inflammatory niche

AD is characterized by a prominent epidermal barrier-alarmin-neuroimmune circuit (2). Barrier disruption, microbial and allergen proteases, scratching injury, keratinocyte-derived TSLP, IL-33 and IL-25 combine with type 2 cytokines, sensory nerve activation, and mast-cell/immune-cell crosstalk to create a self-sustaining inflammatory niche (2, 17). This multicellular network involves keratinocytes, diverse T-cell programs, group 2 innate lymphoid cells, dendritic cells, eosinophils, and other myeloid cells, with their relative contributions varying across disease stages and endotypes (16, 17).

Within this broader network, mast cells may be preferentially conditioned toward inflammatory and neuroimmune amplification, although this does not exclude classical degranulation, protease release, or vascular effects (34, 35). Human AD skin biopsy studies have demonstrated lesion-associated variation in mast cell numbers and tryptase/chymase phenotypes, increased mast cell-nerve contracts, altered chymase activity accompanied by increased mast cell IL-6 expression, and ultrastructural evidence of active secretion (36–39). A recent transcriptomic study further indicates that cytokine-conditioned human mast cells acquire distinct but overlapping functional programs; projection of these signatures onto AD skin single-cell data suggests heterogeneous IL-4/IL-13-, IFN-γ-, TGF-β-, and IL-33-associated mast cell states (40). These findings support context-dependent mast cell plasticity but do not establish a uniform AD-specific mast cell phenotype or demonstrate that any single mode of secretion predominates.

Rather than being expressed predominantly as an immediate vascular response, mast cell outputs in AD, including tryptase, cytokines, and chemokines, may be preferentially integrated into PAR2-related keratinocyte activation, neuronal sensitization, and neuroimmune itch amplification (34, 35, 41, 42). Severe or chronic AD should not be reduced to a simple shift from type 2 to type 1 inflammation. A more appropriate model is immune broadening on a persistent type 2/Th22 background, with variable contributions from Th1, Th17, IL-1β, TNF, myeloid, microbial, and scratching-related signals (16, 17). Within this multicellular program, mast cell outputs may thereby contribute to chronic eczematous inflammation, excoriation, lichenification, and remodeling. Accordingly, “inflammation-primed” denotes a preferential functional tendency imposed by the AD tissue context, not a fixed mast cell subtype or an absence of degranulation.

## Neuropeptides and PAF: shared inputs, divergent wiring

Neuropeptides illustrate shared biology with divergent tissue output. In both CSU and AD, sensory nerves release SP, calcitonin gene-related peptide (CGRP), vasoactive intestinal peptide (VIP), which contribute to itch, neurogenic inflammation, mast-cell activation, vasodilation, and immune-cell recruitment (37, 43, 44). Serum SP is increased in CSU and correlates with disease activity (25). In AD, by contrast, reported associations between circulating neuropeptides and disease severity have been less consistent (45, 46). This pattern may reflect a more chronic, locally regulated neuroepidermal circuit rather than an acute, circulating biomarker of disease activity (17).

The functional wiring differs between the diseases. In CSU, neuropeptides integrate into the dermal neuro-mast cell/vascular axis, particularly through SP-MRGPRX2 signaling (43, 47). MRGPRX2-expression has also been reported in AD skin, and experimental evidence suggests that MRGPRX2-mediated tryptase release can amplify type 2 cytokine production (41). These findings indicate that this pathway may also be incorporated into the chronic inflammatory network of AD, although its cellular distribution, endotype specificity, and relative contribution to human disease remain incompletely defined. Thus, although the receptor pathway is shared, its tissue-level consequences may differ according to the surrounding cellular network.

The same principle applies to coagulation, complement, protease/PAR signaling, and lipid mediators. In CSU, coagulation factor activation, thrombin and plasmin generation, fibrin turnover, and C5a/C5aR signaling are anatomically linked to the vascular/perivascular compartment, facilitating mast cell and basophil activation and endothelial leakage in the setting of increased coagulation potential (15). However, the precise enzymatic mechanisms linking the coagulation, fibrinolysis, and complement cascades remain debated (48). In AD, protease-related and potentially coagulation-related signals may instead operate within a barrier-disrupted epidermal compartment through PAR2-expressing keratinocytes and sensory nerves (17, 41, 49). Within this context, their effects may be preferentially expressed as itch, epithelial inflammation, and remodeling rather than predominantly as vascular leakage. This proposed relative weighting does not imply that TF, PAR2, C5a, PAF, or histamine is assigned exclusively to either disease. Rather, we propose that their tissue-level effects are predominantly channeled through a dermal mast cell-endothelial leakage circuit in CSU or a neuroepidermal inflammatory circuit in AD.

PAF may act in CSU through a vascular/perivascular route that does not necessarily require detectable mast-cell degranulation, although focal mast-cell amplification may occur under selected experimental or disease-related conditions (32, 33). In AD, elevated circulating PAF has been reported, but its cellular routing and tissue-level consequences remain poorly defined (42, 50).

## Network plasticity between AD and CSU

The network framework may also accommodate temporal plasticity between the two diseases. This extension remains speculative. One possibility is that a history of AD may leave a residual type 2/mast cell-primed milieu after overt eczema resolves, sustained by persistently elevated IgE, autoreactive IgE repertoires, eosinophil-biased inflammation, altered lipid mediator tone, neuroimmune sensitization, and residual barrier-immune memory. A second insult, such as autoimmunity, infection, stress, hormonal change, increase of coagulation potential, or IgE repertoire shift, may later shift this primed state toward dermal vascular whealing (1, 11).

The converse may also occur. In some patients with long-standing or treatment-refractory CSU, wheals are incompletely suppressed by antihistamines or omalizumab. This can allow residual tissue inflammation to become more visible. Contributing signals may include IL-13, IL-1β/IL-17A, eosinophilic or myeloid activation, PAF-related signaling, and scratching, consistent with the IL-13-dominant endotype we previously identified in patients with delayed or absent response to omalizumab (12, 51). This endotype is characterized by relatively low IL-33 and a cytokine network that remains largely unchanged even after 6 months of treatment (51). Some patients may therefore acquire AD-like or eczematous inflammatory features. This does not mean that CSU becomes AD, but that the dominant tissue-response program may shift from vascular leakage toward tissue-retained inflammatory itch.

## Future directions

Several predictions are testable. First, longitudinal registries should characterize the bidirectional temporal sequences of AD and CSU and serially assess validated disease-activity indices in patients with both conditions, using strict operational definitions, remission windows, diagnostic validation, and control for surveillance bias. Second, spatial profiling of CSU wheals and AD lesions should map mast cells, basophils, eosinophils, sensory nerves, endothelial activation, PAFR, TF/FVIIa-related coagulation signals, C5a/C5aR, PAR2, keratinocyte alarmins, and histamine or histamine-metabolizing pathways. Third, functional assays should test patient serum-primed mast cells, basophils, endothelial cells, and keratinocytes in parallel. Such systems could determine whether CSU sera preferentially induce endothelial permeability and mast-cell degranulation, whereas AD sera preferentially induce keratinocyte alarmins, cytokines, and neuroepidermal itch mediators. Finally, kinetic approaches to histamine, cytokines and PAF biology are needed, because transient bursts, local dilution, and mediator dispersion may be missed by static measurements.

## Conclusion

CSU and AD are not biologically incompatible diseases. They may represent different tissue interpretations of overlapping IgE-mast cell, neuropeptide, lipid mediator, protease, complement, and type 2 inflammatory signals. CSU is organized around a feedback-limited dermal neuropeptide-mast cell-vascular program that produces transient wheals and angioedema. AD is organized around a disrupted barrier and a multicellular neuro-epidermal cytokine network that favors chronic eczematous inflammation. This tissue address may not be fixed. Priming from prior AD and incomplete control of CSU both suggest that the dominant program can shift over time. Distinguishing shared mediators from their tissue address offers a testable framework for the clinical impression that wheals and eczema rarely reach peak severity together, and may guide biomarker discovery, endotype classification, and mechanism-based treatment selection, pending direct longitudinal and mechanistic validation.

## Data Availability

No new data were generated or analyzed in this study, further inquiries can be directed to the corresponding author/s.

## References

[1] Zuberbier T Ansari ZAH Abdul Latiff AH Abuzakouk MM Agcaoili-De Jesus MS Agondi RC. The international guideline for the definition, classification, diagnosis and management of urticaria. Allergy. (2026) 81:2582–632. 10.1111/all.70210 41649409 PMC13466004

[2] Langan SM Irvine AD Weidinger S. Atopic dermatitis. Lancet. (2020) 396:345–60. 10.1016/S0140-6736(20)31286-1 32738956

[3] Kocatürk E Grattan C Cherrez I Cho YT De A Demir S. The overall burden of chronic spontaneous urticaria—the current landscape. Br J Dermatol. (2026). 10.1093/bjd/ljag121 41913980

[4] Cortes JA Adjei-Frimpong NA Fakhouri SK Delacqua F Oldenburg R. Atopic comorbidities associated with chronic spontaneous urticaria: a case-control analysis of the all of US research program. JEADV Clin Pract. (2026) 5:194–7. 10.1002/jvc2.70181 41809919 PMC12969763

[5] Fukunaga A Kishi Y Sunaga Y Arima K. Prevalence, comorbidities, and current management of chronic spontaneous urticaria in Japan: retrospective claims database study. J Dermatol. (2025) 52:1775–87. 10.1111/1346-8138.17943 40922384 PMC12698888

[6] Toubi E Mubariki R Vadasz Z. Co-existence of chronic spontaneous urticaria with atopic dermatitis: clinical and immunological perspectives. Front Allergy. (2025) 6:1681375. 10.3389/falgy.2025.1681375 41368041 PMC12682838

[7] Chiu HY Muo CH Sung FC. Associations of chronic urticaria with atopic and autoimmune comorbidities: a nationwide population-based study. Int J Dermatol. (2018) 57:822–9. 10.1111/ijd.14000 29663342

[8] Ghazanfar MN Kibsgaard L Thomsen SF Vestergaard C. Risk of comorbidities in patients diagnosed with chronic urticaria: a nationwide registry-study. World Allergy Organ J. (2020) 13:100097. 10.1016/j.waojou.2019.100097 32021661 PMC6994395

[9] Kitsioulis NA Papadopoulos NG Kostoudi S Manousakis E Douladiris N Xepapadaki P. Assessment of atopic dermatitis as a risk factor for chronic spontaneous urticaria in a pediatric population. Allergy Asthma Proc. (2018) 39:445–8. 10.2500/aap.2018.39.4166 30401321

[10] Xiao M Zhang X Guo A Zhang K Zhao Q Fan L. A retrospective cohort study on the comorbidity in 19,264 Chinese patients of different ages with urticaria. Acta Derm Venereol. (2024) 104:adv24050. 10.2340/actadv.v104.24050 38932592 PMC11223577

[11] Kolkhir P Bonnekoh H Metz M Maurer M. Chronic spontaneous urticaria: a review. J Am Med Assoc. (2024) 332:1464–77. 10.1001/jama.2024.15568 39325444

[12] Ye YM. Targeting immune dysregulation in chronic spontaneous urticaria: beyond antihistamines. Korean J Intern Med. (2026) 41:418–31. 10.3904/kjim.2025.374 42128382 PMC13172814

[13] Lerner L Babina M Zuberbier T Stevanovic K. Beyond allergies—updates on the role of mas-related G-protein-coupled receptor X2 in chronic urticaria and atopic dermatitis. Cells. (2024) 13:220. 10.3390/cells13030220 38334612 PMC10854933

[14] Chovatiya R Hawkes JE DiRuggiero D Pansch LA Simcox E Gonzalez T. Type 2 inflammation and its role in dermatologic diseases. Int J Dermatol. (2025) 64:978–91. 10.1111/ijd.17707 40119613 PMC12082622

[15] Yanase Y Takahagi S Ozawa K Hide M. The role of coagulation and complement factors for mast cell activation in the pathogenesis of chronic spontaneous urticaria. Cells. (2021) 10:1759. 10.3390/cells10071759 34359930 PMC8306267

[16] Fyhrquist N Yang Y Karisola P Alenius H. Endotypes of atopic dermatitis. J Allergy Clin Immunol. (2025) 156:24–40.e4. 10.1016/j.jaci.2025.02.029 40054744

[17] Weidinger S Aoki V Dhar S Dlova NC Kabashima K Ma L. Atopic dermatitis. Nat Rev Dis Primers. (2026) 12:36. 10.1038/s41572-026-00703-z 42243136

[18] Kolkhir P Muñoz M Asero R Ferrer M Kocatürk E Metz M. Autoimmune chronic spontaneous urticaria. J Allergy Clin Immunol. (2022) 149:1819–31. 10.1016/j.jaci.2022.04.010 35667749

[19] Tbakhi B Ware K Park HS Bernstein JS Bernstein JA. An overview of chronic spontaneous urticaria: diagnosis, management, and treatment. Allergy Asthma Immunol Res. (2025) 17:531–46. 10.4168/aair.2025.17.5.531 41044830 PMC12511800

[20] Andrades E Clarós M Torres JV Nonell L González M Curto-Barredo L. New transcriptome and clinical findings of platelet-activating factor in chronic spontaneous urticaria: pathogenic and treatment relevance. Biofactors. (2022) 48:1284–94. 10.1002/biof.1880 35927787

[21] Petersen LJ Church MK Skov PS. Histamine is released in the wheal but not the flare following challenge of human skin in vivo: a microdialysis study. Clin Exp Allergy. (1997) 27:284–95. 10.1046/j.1365-2222.1997.d01-502.x 9088655

[22] Seirin-Lee S Matsubara D Yanase Y Kunieda T Takahagi S Hide M. Mathematical-based morphological classification of skin eruptions corresponding to the pathophysiological state of chronic spontaneous urticaria. Commun Med. (2023) 3:171. 10.1038/s43856-023-00404-8 38049619 PMC10696082

[23] Seirin-Lee S Takahagi S Hide M. Pathophysiological mechanisms of the onset, development, and disappearance phases of skin eruptions in chronic spontaneous urticaria. Bull Math Biol. (2025) 87:1. 10.1007/s11538-024-01380-3 PMC11564288 39541050

[24] Zuberbier T Bonnekoh H Kocatürk E Kolkhir P Lionnet L Muñoz M. Insights into pathogenesis of chronic spontaneous urticaria. Br J Dermatol. (2026). 10.1093/bjd/ljag277 42455161

[25] Metz M Krull C Hawro T Saluja R Groffik A Stanger C. Substance P is upregulated in the serum of patients with chronic spontaneous urticaria. J Invest Dermatol. (2014) 134:2833–6. 10.1038/jid.2014.226 24844859

[26] Varricchi G Pecoraro A Loffredo S Poto R Rivellese F Genovese A. Heterogeneity of human mast cells with respect to MRGPRX2 receptor expression and function. Front Cell Neurosci. (2019) 13:299. 10.3389/fncel.2019.00299 31333418 PMC6616107

[27] Oskeritzian CA Zhao W Min H-K Xia H-Z Pozez A Kiev J. Surface CD88 functionally distinguishes the MCTC from the MCT type of human lung mast cell. J Allergy Clin Immunol. (2005) 115:1162–8. 10.1016/j.jaci.2005.02.022 15940129 PMC1460014

[28] Gibbs BF Wierecky J Welker P Henz BM Wolff HH Grabbe J. Human skin mast cells rapidly release preformed and newly generated TNF-alpha and IL-8 following stimulation with anti-IgE and other secretagogues. Exp Dermatol. (2001) 10:312–20. 10.1034/j.1600-0625.2001.100503.x 11589728

[29] Okayama Y Ono Y Nakazawa T Church M Mori M. Human skin mast cells produce TNF-by substance P. Int Arch Allergy Immunol. (1998) 117:48–51. 10.1159/000053571 9758897

[30] Schroeder JT MacGlashan DW Jr Kagey-Sobotka A White JM Lichtenstein LM. IgE-dependent IL-4 secretion by human basophils. The relationship between cytokine production and histamine release in mixed leukocyte cultures. J Immunol. (1994) 153:1808–17. 10.4049/jimmunol.153.4.1808 7519213

[31] Eglite S Pluss K Dahinden CA. Requirements for C5a receptor-mediated IL-4 and IL-13 production and leukotriene C4 generation in human basophils. J Immunol. (2000) 165:2183–9. 10.4049/jimmunol.165.4.2183 10925305

[32] Krause K Giménez-Arnau A Martinez-Escala E Farré-Albadalejo M Abajian M Church MK. Platelet-activating factor (PAF) induces wheal and flare skin reactions independent of mast cell degranulation. Allergy. (2013) 68:256–8. 10.1111/all.12083 23320562

[33] Choi BY Yang E-M Jung H-W Shin M-K Jo J Cha H-Y. Anti-heat shock protein 10 IgG in chronic spontaneous urticaria: relation with miRNA-101-5p and platelet-activating factor. Allergy. (2023) 78:3166–77. 10.1111/all.15810 37415527

[34] Xiao Z Zhuo Y Li R Tan Y. The recent advances of mast cells in the pathogenesis of atopic dermatitis. Front Allergy. (2025) 6:1668742. 10.3389/falgy.2025.1668742 41099016 PMC12518229

[35] Li D Han Y Zhou J Yang H Chen J Tey HL. Mast cell–neuron axis as a core mechanism in chronic pruritus of atopic dermatitis: from mechanistic insights to therapeutic targets. Front Immunol. (2025) 16:1645095. 10.3389/fimmu.2025.1645095 41235218 PMC12604997

[36] Jarvikallio A Naukkarinen A Harvima IT Aalto ML Horsmanheimo M. Quantitative analysis of tryptase- and chymase-containing mast cells in atopic dermatitis and nummular eczema. Br J Dermatol. (1997) 136:871–7. 10.1046/j.1365-2133.1997.01831.x 9217819

[37] Jarvikallio A Harvima IT Naukkarinen A. Mast cells, nerves and neuropeptides in atopic dermatitis and nummular eczema. Arch Dermatol Res. (2003) 295:2–7. 10.1007/s00403-002-0378-z 12709813

[38] Zhang B Alysandratos K-D Angelidou A Asadi S Sismanopoulos N Delivanis D-A. Human mast cell degranulation and preformed TNF secretion require mitochondrial translocation to exocytosis sites: relevance to atopic dermatitis. J Allergy Clin Immunol. (2011) 127:1522–31. 10.1016/j.jaci.2011.02.005 21453958 PMC3381794

[39] Ilves T Harvima IT. Decreased in chymase activity is associated with increase in IL-6 expression in mast cells in atopic dermatitis. Act Derm Venereol. (2015) 95:411–6. 10.2340/00015555-1979 25270893

[40] Tontini C Bahri R Higham A Singh D Simpson A Bulfone-Paus S. Microenvironment-driven mast cell plasticity: insights from cytokine-activated gene signatures in skin and respiratory diseases. Allergy. (2025) 80:3077–94. 10.1111/all.70052 40926620 PMC12590347

[41] Jia T Che D Zheng Y Zhang H Li Y Zhou T. Mast cells initiate type 2 inflammation through tryptase released by MRGPRX2/MRGPRB2 activation in atopic dermatitis. J Invest Dermatol. (2024) 144:53–62.e2. 10.1016/j.jid.2023.06.201 37482287

[42] Wong LS Yen YT Lee CH. The implications of pruritogens in the pathogenesis of atopic dermatitis. Int J Mol Sci. (2021) 22:7227. 10.3390/ijms22137227 34281281 PMC8269281

[43] Aitella E Bruno M Azzellino G De Martinis M Ginaldi L Romano C. Mast cells and substance P: neuroinflammatory loops at the molecular and translational clinical levels. Biomolecules. (2026) 16:539. 10.3390/biom16040539 42072660 PMC13113954

[44] Shi Z Deng X Ren J Lu S Tan G. Serum neuropeptides in chronic spontaneous urticaria: associations with disease activity and clinical phenotypes. Allergy Asthma Immunol Res. (2026) 18:453–60. 10.4168/aair.2026.18.3.453 42223042 PMC13226869

[45] Paramita DA Nasution K Lubis NZ. Relationship of substance P with the degree of atopic dermatitis severity. Clin Cosmet Investig Dermatol. (2021) 14:551–5. 10.2147/ccid.S306557 34079320 PMC8164664

[46] Salomon J Baran E. The role of selected neuropeptides in pathogenesis of atopic dermatitis. J Eur Acad Dermatol Venereol. (2008) 22:223–8. 10.1111/j.1468-3083.2007.02399.x 18211417

[47] Fujisawa D Kashiwakura J Kita H Kikukawa Y Fujitani Y Sasaki-Sakamoto T. Expression of mas-related gene X2 on mast cells is upregulated in the skin of patients with severe chronic urticaria. J Allergy Clin Immunol. (2014) 134:622–33. 10.1016/j.jaci.2014.05.004 24954276

[48] Sakurai Y. Reframine ATR: from the immunologic/nonimmunologic dichotomy to adaptive, innate, and physicochemical drivers within the allergy-inflammation-coagulation axis. Front Immunol. (2026) 17:1892622. 10.3389/fimmu.2026.1892622 42756170 PMC13582587

[49] Redhu D Franke K Aparicio-Soto M Kumari V Pazur K Illerhaus A. Mast cells instruct keratinocytes to produce thymic stromal lymphopoietin: relevance of the tryptase/protease-activated receptor 2 axis. J Allergy Clin Immunol. (2022) 149:2053–61.e6. 10.1016/j.jaci.2022.01.029 35240143

[50] Gomułka K Wójcik E Szepietowski JC. Serum levels of eosinophil-derived neurotoxin, platelet-activating factor and vascular endothelial growth factor in adult patients with atopic dermatitis—a pilot study. Biomedicines. (2022) 10:3109. 10.3390/biomedicines10123109 PMC9775762 36551865

[51] Ye Y-M Lee H-Y Jang J-H Moon D-H Park K-W Park S. Cytokine imbalance and immune network endotypes underlying heterogeneous response to omalizumab in chronic spontaneous urticaria. Front Immunol. (2026) 17:1783483. 10.3389/fimmu.2026.1783483 42088522 PMC13135958

